# Lipoic acid inhibits cognitive impairment induced by multiple cell phones in young male rats: role of Sirt1 and Atg7 pathway

**DOI:** 10.1038/s41598-023-44134-2

**Published:** 2023-10-28

**Authors:** Bataa M. A. El-Kafoury, Enas A. Abdel-Hady, Wesam El Bakly, Wael M. Elayat, Ghada Galal Hamam, Samar M. M. Abd El Rahman, Noha N. Lasheen

**Affiliations:** 1 Department of Medical Physiology, Faculty of Medicine, Ain Shams University, Cairo, Egypt; 2 Department of Clinical Pharmacology, Faculty of Medicine, Ain Shams University, Cairo, Egypt; 3 Department of Medical Pharmacology, Faculty of Medicine, AFCM, Cairo, Egypt; 4 Department of Biochemistry and Molecular Biology, Faculty of Medicine, Ain Shams University, Cairo, Egypt; 5 Department of Basic Medical Sciences, Faculty of Medicine, Galala University, Galala City, Egypt; 6 Department of Histology and Cell Biology, Faculty of Medicine, Ain Shams University, Cairo, Egypt; 7 Department of Basic Medical Sciences, Faculty of Medicine, Galala University, Galala City, Egypt

**Keywords:** Biochemistry, Cell biology, Molecular biology, Neuroscience, Physiology

## Abstract

The utilization of digital technology has grown rapidly in the past three decades. With this rapid increase, cell phones emit electromagnetic radiation; that is why electromagnetic field (EMF) has become a substantial new pollution source in modern civilization, mainly having adverse effects on the brain. While such a topic attracted many researchers’ scopes, there are still minimal discoveries made regarding chronic exposure to EMF. The extensive use of cell phones may affect children's cognition even indirectly if parents and guardians used their phones repeatedly near them. This study aims to investigate possible lipoic acid (LA) effects on cognitive functions and hippocampal structure in young male rats exposed to electromagnetic fields (EMF) emitted from multiple cell phones. Forty young male Wistar rats were randomly allocated into three groups: control, multiple cell phones-exposed and lipoic acid-treated rats. By the end of the experimental period, the Morris water maze was used as a cognitive test. The rats were sacrificed for the collection of serum and hippocampal tissue. These serum samples were then utilized for assessment of Liver function tests. The level ofglutamate, acetylcholine (Ach) and malondialdehyde (MDA) was estimated, in addition to evaluating the expression of autophagy-related protein-7 (Atg7) and Sirt1 genes. The left hippocampal specimens were used for histopathological studies. Results showed that multiple cell phone-exposed rats exhibited shorter latency time to reach the platform by the fifth day of training; additionally, there was a reduction in consolidation of spatial long-term memory. Correspondingly, there was an elevation of hippocampal Ach, glutamate, and MDA levels; accompanied by up-regulation of hippocampal Sirt1 and Atg7 gene expression. Compared to the EMF-exposed group, LA administration improved both learning and memory, this was proved by the significant decline in hippocampal MDA and Ach levels, the higher hippocampal glutamate, the downregulated hippocampal Sirt1 gene expression and the upregulated Atg7 gene expression. In conclusion, EMF exposure could enhance learning ability; however, it interfered with long-term memory consolidation shown by higher hippocampal Ach levels. Lipoic acid treatment improved both learning and memory by enhancing autophagy and hippocampal glutamate level and by the reduced Ach levels and Sirt1 gene expression.

## Introduction

The global spread of using cell phones at variable age groups has raised concerns about health risks due to the emitted EMR. Children get exposed to cell phones EMF as young as four years old; therefore, it is mandatory to conduct evidence-based research to highlight and demonstrate the negative impacts of cell phones on health. Even though the wave absorption of a child’s skull is double that of an adult, the absorption of the skull bone marrow in children is ten times more than that of adults. Phone placement is a contributing factor as cognitive function is affected due to the cell phone’s position near the user’s head^1,2^.

Mobile phones' EMR, absorbed by the body, especially the head region, could influence cognitive function in humans through their thermal or non-thermal effect on protein production^3^.

Controversial studies about the negative impact of EMF on health and cognition were conducted over the past years; this raised a two-sided debate that has been going on in this field. To illustrate, Luria et al.^4^ demonstrated changes in a spatial working memory task in terms of the response time and accuracy in humans exposed to cell phone EMF. It has been then documented that those rats exposed to cell phone EMF for 4 weeks had delayed learning as well as morphological changes in the CA3 region of the hippocampus^5^.

In contrast, extremely low-frequency electromagnetic fields, 12 Hz, were proven to improve memory and cognitive functions, as shown by the increasing visual working memory test scores^6^.

Low-level reactive oxygen species (ROS) play a critical role as second messengers, triggering signaling cascades, which regulate cell proliferation, apoptosis, and other key cellular processes. However, uncontrolled ROS production from cell phones EMF exposure can lead to oxidative damage to membrane phospholipids, nucleic acids, and proteins, which can disrupt normal cellular processes and trigger severe cell dysfunctions^7,8^. Cognitive impairment and pyramidal neuron loss are consequences of brain tissue damage^9^.

Given the close correlation between the production of ROS and mitochondrial function, an interest is emerging in the role of such organelles in the responses evoked by EMF insult in biosystems. Gupta et al.^10^ demonstrated that four week—cell phone exposure in rats caused loss of mitochondrial function and integrity, with enhanced cytochrome C, caspase-3, and caspase-9 activities in the hippocampus.

Apart from ATP production, mitochondria have a role in cell signaling via ROS production, affecting cell growth and differentiation in addition to programmed cell death in normal signaling^11^. The mitochondrion is a self-autonomous double membranous organelle found in most eukaryotic organisms^12^. The half-life period of mitochondria is about 2–3 weeks. Mitochondria are in a dynamic state in the cells; fission–fusion^13^. This continual state of fission and fusion is a key flagging identity of the mitochondrion in a transient condition^14^. At any point in the cell cycle, a mitochondrion may undergo fission to give rise to two separate mitochondria^15^. Simultaneously mitochondria are undergoing fusion in which both the inner and outer membranes of the mitochondria break and rejoin to form a single intact mitochondrion. Fission and fusion events are governed by the mitochondrial shape transition due to unknown mechanisms during cellular activation^16^.

In pathological conditions, there is a shift toward fission and inhibition of autophagy with subsequent loss of mitochondrial networks and accumulation of dysfunctional mitochondria in the cell, thereby enhancing ROS production and impairing ATP production^17^. Thus, mitophagy (mitochondrial autophagy) protects injured tissues in various diseases through the removal and recycling of damaged mitochondria^18^.

Therefore, autophagy may protect the neuronal cell bodies in the cerebral cortex during cell phone-EMF exposure in mice. Meanwhile, neuronal cell bodies were still structurally stable except for demyelination which was accompanied by subsequent neurological and/or neurobehavioral disorders^19^.

Autophagy, a process of degradation of cell constituents including intracellular organelles, is a protective cellular response to stress to remove aged or dysfunctional macromolecules and organelles to maintain cellular homeostasis. It occurs normally at low levels and could be accelerated by cellular stressors such as nutrient starvation, DNA damage and organelle damage^20,21^. More than 30 autophagy-related proteins (autophagy-specific gene, Atgs) may drive such a process. Atg5 and Atg7 were reported to be essential molecules for autophagy^18^.

In addition, alpha-Lipoic acid (LA) had an important neuroprotective effect^22^; it was found that LA through its strong antioxidant effects caused improvement in cognitive dysfunction^23^. Accordingly, it is valuable to investigate the effects of LA supplementation as a treatment of brain changes after cell phone exposure. Little information is present about the possible neuroprotective effects of lipoic acid after EMF-cell phone exposure from multiple sources.

The authors were the first to demonstrate the beneficial effects of LA on EMF-induced cognitive changes in young rats in a previous study using single cell phone exposure^24^. However, up to the authors’ knowledge, no previous study investigated such changes in the puberty and adolescence transition period using EMF emitted from multiple cell phones, in a trial to simulate the higher number of cell phones used by the family members within the same place.

### Aim of the work

This study was designed to investigate the effects of lipoic acid supplementation on brain function and structure in young rats after long-term exposure to EMF emitted from multiple cell phones and to explore and aaddressthe possible underlying mechanisms.

## Materials and methods

### Animals

This study was performed on 40 young male Wistar rats, initially weighing 70–90 g (6 weeks old), purchased from Animal Farm (El-Zyad import office of experimental animals for colleges and research centers), Giza. Rats were housed in the Medical Ain Shams Research Institute (MASRI), Faculty of Medicine, Ain Shams University, under standard conditions of boarding at room temperature 22–25 °C, normal light–dark cycle and free access to food and water- ad libitum. All animal experiments were performed according to the National Institutes of Health guide for the care and use of Laboratory animals (NIH) Publications No. 8023, updated in 2011 (8th edition). Also, the Ain Shams Faculty of Medicine Ethical Committee approval, in Egypt, was obtained (MS331). This study is reported following ARRIVE guidelines.

### Chemicals and drugs

Alpha lipoic acid (LA) was purchased from Sigma Aldrich Chemicals, Germany, which was supplied as a powder. The sodium salt of the LA solution was prepared by dissolving one gram of LA and 40 ml of 1 M NaOH. Then, this aqueous suspension was neutralized with 1 M HCl to obtain a completely water-soluble form^25^.

### Experimental protocol

Rats were randomly allocated into three groups as follows:

Group I: Control group (n = 14): They were transferred to cages of EMF exposure while the cell phone is switched off. They were further subdivided into:

- Negative Control subgroup (n = 7): They received intraperitoneal (i.p.) injection of normal saline solution (0.9% NaCl) in an equivalent volume to LA solution during the last 3 weeks of EMF exposure.
- Positive Control subgroup (n = 7): They were supplemented with LA in the same dose as the LA-treated group during the last 3 weeks of EMF exposure.

Both subgroups were pooled together due to the absence of any different results in varied parameters, assayed in the current study.

Group II: Cell Phones-Exposed group (n = 13): Rats in this group were exposed to electromagnetic fields emitted from three cell phones, two hours/day, six days/week for 12 weeks^26^.

Group III: Lipoic acid (LA)-treated Cell Phone-Exposed group (n = 13): They were exposed to an electromagnetic field similar to group II and received i.p. injection of LA in a dose of 50 mg/kg^27^ for the last 3 weeks of exposure duration, from week 10 till week 12.

### Cell phones-electromagnetic field exposure technique

Both groups II and III were exposed to a radiofrequency field emitted from three cell phones, using Global System for Mobile Communication (GSM) cell phone, LG B220. The GSM cell phones operate with microwave carrier frequencies in the GHz range (850–1900 MHz)^28^. The EMF exposure was performed at a fixed time daily (from 10 to 12 A.M.). Rats had sufficient free space for propagation of EMF, in addition to free access to food and water.

The exposure was carried out in plexiglass cages (20 × 31 × 13.5 cm), three rats per cage, and cell phones were placed under the cage at a 0.5 cm distance^29^. All three cell phones were distributed down the cage at its midline. The exposed groups were irradiated with 50 missed calls/hour (missed call duration 35 s) separated by 15-s intervals on the silent non-vibratory mode per day^5^. They were kept in the silent non-vibratory mode to prevent any stress and disturbing effects of the background sounds or ring tone-induced vibrations on animal learning and memory during the time of exposure^30^. Rats in all groups were free to move during the exposure period to cancel the effect of immobilization.

The intensity of electromagnetic fields emitted from cell phones was 4.8milliGauss (10^–7^ Tesla) at the center of the exposure cage with minimal changes at the corners, as measured by Gauss/Teslameter, 4048, USA (Courtesy of Faculty of Science, Ain Shams University).

### Experimental procedures

At the end of the experimental period (12 weeks), all rats were subjected to a behavioral test (cognitive function test).

A) Morris water maze training
  The behavioral training and testing were conducted in a water maze, which was a brown circular pool (180 cm in diameter and 50 cm high) filled to a 25 cm depth with 22 ± 2 °C water^31^. ‘‘Atlantis platform’’ (10 cm diameter) was at the center of one quadrant (North-East quadrant) of the maze and submerged 3 cm below the surface of the water. Water was rendered opaque by the addition of powdered milk and the maze was in a room containing several constant, salient visual cues (posters, objects, and equipment). A video camera was mounted on the ceiling directly above the pool to record the swim path of each rat.
  In the spatial training procedure, rats received four trials in the Morris water maze on each of the five days of training^32^. On each trial, the rat was placed into the water at one of the four cardinal points of the compass (N, E, S, W), which varied from trial to trial in a quasi-random order. The rat had to swim until it climbed onto the escape platform. The latency was recorded till the rat reached the platform and remained on it for 10 s. If the rat did not reach the platform within 120 s, the trial was terminated, and the rat was placed on the platform for 10 s. Thereafter, rats were transferred to a dry holding cage where they remained for 60 s until the next trial. After training, rats were returned to their home cages.
  On day 6, rats were returned to the water maze for a retention test. They were given a 60-sec probe trial during which the platform was removed. The parameter measured from the probe test was the time spent (%) in the target quadrant of the task.
B) Animal sacrifice
  On the day of sacrifice (next to performing the Morris water maze test), overnight fasting rats were weighed and anaesthetized with an i.p. injection of Pentobarbitone (40 mg/kg B.W.).
C) Tissue collection
  When the stage of surgical anesthesia was reached, this was checked by loss of withdrawal reflexes, and a midline abdominal incision was made. Abdominal aorta was cannulated to collect blood samples. Separated plasma samples were used for the determination of liver function tests to exclude liver failure-induced rise of glutamate in brain tissues.

Then, the rat skull was opened, and the brain was dissected out and washed with saline. The brain was divided at the midsagittal plane into right and left hemispheres, and the hippocampus was reached from the medial side. Thereafter, the whole brain stem and cerebellum were removed^33^. The right hippocampi from all rats were stored at − 80 °C for subsequent determination of glutamate and acetylcholine (Ach), MDA and some mitochondrial-related gene expression.

Glutamate neurotransmitter was determined in the hippocampus by ultra-pressure liquid chromatography (UPLC), while acetylcholine was assayed in the hippocampus by choline competitive-ELISA method supplied by MyBioSource, USA. Right hippocampal tissue MDA level was measured according to Satoh^34^ and Ohkawa et al.^35^, using colorimetric kits supplied by Bio-diagnostic, Egypt.

Determination of plasma Aspartate Aminotransferase (AST) and Alanine Aminotransferase (ALT) activities were carried out by U.V. kinetic method, using kits supplied by spectrum-diagnostic, Egypt. They were performed to exclude any brain changes induced by liver function alterations after EMF exposure.

### RNA isolation from right hippocampal tissue and qRT-PCR

Equally weighed frozen right hippocampal tissues were homogenized followed by a total RNA extraction using TRIzol reagent (Invitrogen) (Fischer Scientific cat no 15596026). Then, the concentration and purity of RNA were determined by NanoDrop 1000 spectrophotometer. For cDNA synthesis, one µg of RNA was reverse transcribed using QuantiTect® Reverse Transcription kit (QIAGEN, Germany) (Cat no. 205311).

Quantitative Real-time PCR (qRT-PCR) was performed for Sirt1 and Atg7 genes using Fast SYBR Green master mix (Qiagen Germany) (Cat no. 204141), and 5 plex rotor gene™ system (Qiagen) according to manufacturer's instructions.

The primers for Sirt1 were designed and purchased from Macrogen, (Seoul, Korea). Sirt1 primers sequence were (F: TGTAGATGAGGCAGAGGTTCCC and R: ATCAGGTAGTTCCTCGGTGTCC), meanwhile, Atg7 was purchased from Qiagen (Germany), in the form of SG quantiTect primer (cat no. 249900; Assay ID: QT0008974). The reference gene was the B-actin gene, purchased from Qiagen (Germany) (HS_ACTB_1 quantiTect primer; cat no. 249900; Assay ID: QT000954 31). The cyclic conditions of QuantiTect SYBR Green PCR are shown in Table 1.

**Table 1:** Cycling Conditions of QuantiTect SYBR Green PCR.

Step	Time	Temperature
PCR initial activation step	15 min	95 °C
Step cycling		
Denaturation	15 s	94 °C
Annealing	30 s	50–60 °C
Extension	15 s	72 °C
Number of cycles	35–45 cycles	

The target gene expressions were defined based on the cycle threshold (C_t_), where their expression levels were calculated as ${2}^{{ - \Delta \Delta {\text{C}}_{{\text{t}}} }}$ after normalization to the relative expression of the β-actin gene^36^.

### Histopathological studies

Left hippocampi were immediately fixed in 10% formalin for seven days followed by dehydration in ascending grades of ethyl alcohol, for clearing. Thereafter, they were embedded in paraffin. Paraffin sections were cut at 4–6 µm, and were stained with the following stains:

1. Hematoxylin and Eosin (H&E) stain according to Suvarna et al.^37^.
2. Toluidine blue stain for demonstration of Nissl’s granules, which appeared as a blue reaction in the cytoplasm of nerve cells according to Suvarna et al.^37^.
3. Immunohistochemical stain
  Paraffin sections were cut on positively charged slides and were stained with Avidin Biotin peroxidase technique to detect Glial fibrillary acidic protein (GFAP). This stain is specific for the intermediate filaments fibrillary acidic protein found in astrocytes. Anti-GFAP antibody Ab-1 (clone GA-5), a mouse monoclonal IgG antibody, was purchased from Thermo Scientific, USA. In positive GFAP immuno-stained sections, brown reactions appeared in the cytoplasm of astrocytes^37^.

Morphometric analysis was performed in the Department of Histology and Cell Biology, Faculty of Medicine, Ain Shams University, Cairo, Egypt. Image analyzer Leica Q win V.3 program installed on a computer was used. It was connected to a Leica DM2500 microscope (Wetzlar, Germany). Specimens from all rats in each group were subjected to a morphometric study. Measurements were taken from five different slides obtained from each rat. Stained sections of hippocampus from the CA3 region from each group were examined; five high power fields/sections were randomly chosen to measure: Nissl’s granules density in the pyramidal cells’ cytoplasm stained by Toluidine blue and the mean area percentage of astrocytes stained by GFAP immune reaction. Sections were examined using ×40 objective lens (final magnification ×400).

### Statistical analysis

Results, in the present study, were expressed as mean ± SE of the mean. Statistical analysis of the cognitive function test was performed using Graphpad prism, software program, version 5.0 (2007) (GRAPHPAD Inc., CA, USA). The statistical difference among groups was determined using a two-way ANOVA test using Bonferroni's post-Hoc test. To calculate the percentage of time spent in the target quadrant on day 6, the Kruskal–Wallis test was used, followed by Dunn’s multiple comparisons test.

Also, One-Way ANOVA for the difference between means of different groups was performed on obtained results rather than cognitive tests, using post-Hoc test. The Statistical Package for the Social Sciences (SPSS, Inc., Chicago, IL, USA) program, version 20.0, was used. The differences were considered significant when *P* ≤ 0.05.

Correlation Studies were calculated using Pearson's correlation coefficient. The probability of (*P* ≤ 0.05; 2tailed) was considered statistically significant.

## Results

### Behavioral test (Morris Water Maze)

**Figure 1:**
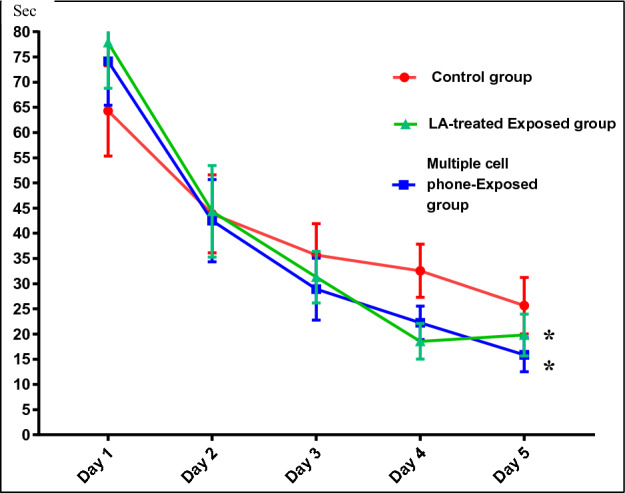
Effects of multiple cell phones- exposure and LA treatment on the performance of spatial learning phase in Morris water maze test (latency to reach target quadrant (sec) in the 5 days of training). *: Significance from the control group by two-way ANOVA at *P* ≤ 0.05.

**Figure 2:**
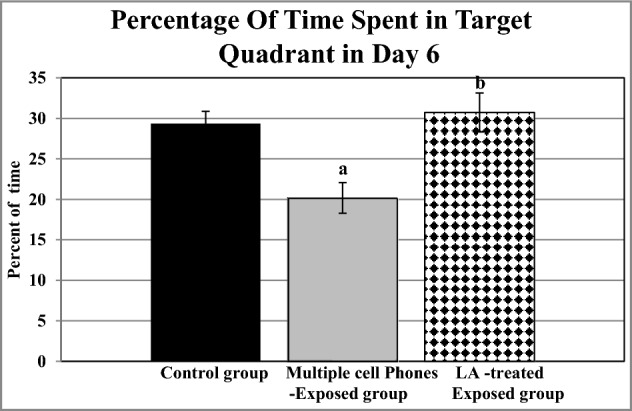
Effects of EMF exposure and lipoic acid treatment on the performance of Morris water maze in the probe trial (percentage of time spent in target quadrant in day 6). a: Significance from the control group by LSD, at *P* ≤ 0.05. b: Significance from multiple cell phone-exposed group by LSD, at *P* ≤ 0.05.

- Latency to Reach Target Quadrant (testing for learning ability)
  As shown in Table 2 and Fig. 1, there was a decline in latency of escape to the platform throughout the five-days of training, this indicates successful training.
  The percentage of change in latency to reach platform between first and fifth day was calculated among the different studied groups (Table 2). Multiple cell phones-exposed rats had a significantly shorter time of latency to reach the platform by 5th day of training (*P* < 0.01). In LA-treated exposed rats, there was a significantly shorter latency compared to the controls, although it did not reach the statistical level of significance when compared to the untreated rats.
- Percentage of Time Spent in Target Quadrant Day 6 (testing for the spatial memory)
  The time spent in the target quadrant in multiple cell phones-exposed group, was significantly lower than that of control. In LA treated-exposed group, it was significantly longer than that of the corresponding untreated group (Table 3 and Fig. 2).

**Table 2:** Changes in Morris Water Maze (latency to reach target quadrant (sec) in the 5 days) and (Percentage of Change of latency in Day 5) in the different studied groups.

	Control group	Multiple cell Phones- Exposed group	LA- Treated-Exposed Group
Day 1			
Mean ± SEM	64.31 ± 8.95	74.15 ± 8.74	77.87 ± 9.08
Day 2			
Mean ± SEM	43.88 ± 7.73	42.5 ± 8.18	44.37 ± 9.1
Day 3			
Mean ± SEM	35.67 ± 6.24	28.92 ± 6.15	31.31 ± 5.12
Day 4			
Mean ± SEM	32.56 ± 5.27	22.23 ± 3.33	18.56 ± 3.53
Day 5			
Mean ± SEM	25.65 ± 5.59	15.88 ± 3.36	19.81 ± 4.13
Percent of change			
Mean ± SEM	− 19.97 ± 15.78	− 78.55 ± 3.21	− 75.52 ± 4.14
a		< 0.01	< 0.05
b			NS

Statistical significance was determined using two-way ANOVA for latency to reach target quadrant in the 5 days and one-way ANOVA for percentage of change of latency in day 5.

a: Significance from the control group.

b: Significance from multiple cell phones- exposed group.

*NS* not significant.

**Table 3:** Changes in Morris Water Maze (% Of Time Spent in Target Quadrant Day 6) in the different studied groups.

	Control group	Multiple cell phones- exposed group	LA- treated-exposed group
Mean ± SEM	30.17 ± 1.09	20.18 ± 1.89	30.73 ± 2.41
a		< 0.01	NS
b			< 0.001

Statistical significance was determined using one-way ANOVA.

a: Significance from the control group.

b: Significance from multiple cell phone-exposed group.

*NS* not significant.

### Biochemical changes

**Figure 3:**
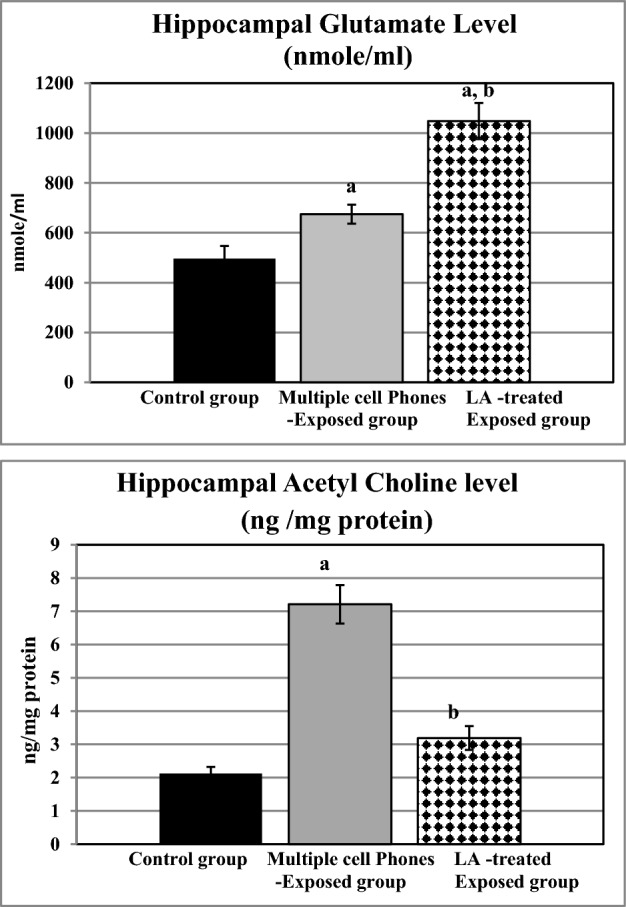
Hippocampal levels of glutamate (nmole /ml) and acetyl choline (ng /mg protein) in the different studied groups. a: Significance from the control group by LSD, at *P* ≤ 0.05. b: Significance from multiple cell phones- exposed group by LSD, at *P* ≤ 0.05.

Hippocampal glutamate level was significantly elevated in both untreated and LA-treated cell phones-exposed rats compared to the controls. LA supplementation caused a significant rise in hippocampal glutamate level compared to the respective untreated group (Fig. 3).

Compared to the control group, hippocampal Ach level was significantly raised in multiple cell phones-exposed rats; meanwhile, it was significantly reduced in LA-treated rats in comparison to the untreated ones (Fig. 3).

**Figure 4:**
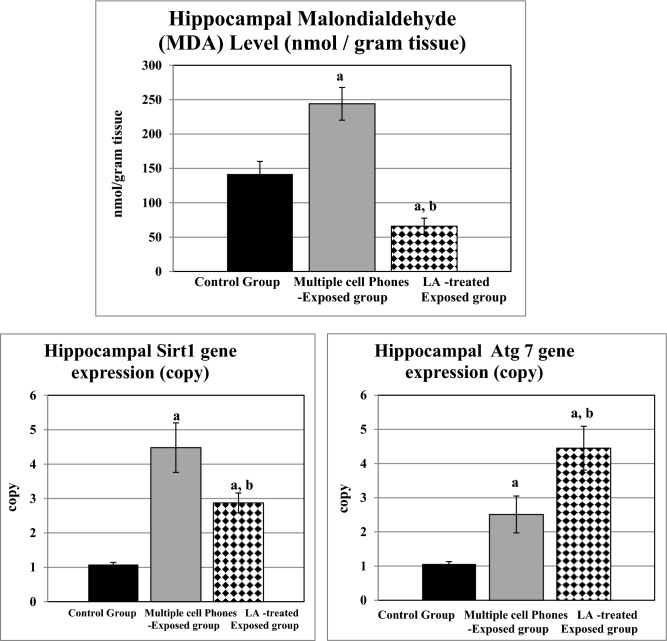
Changes in hippocampal malondialdehyde (MDA) level (nmol/gram tissue) and hippocampal Sirt1 and Atg 7 gene expression (copy) in the different studied groups. a: Significance from the control group by LSD, at *P* ≤ 0.05. b: Significance from multiple cell phones-exposed group by LSD, at *P* ≤ 0.05.

Hippocampal Malondialdehyde (MDA) Level was significantly elevated in the untreated multiple cell phones-exposed group compared to the controls, although it was significantly reduced in LA-treated exposed rats compared to either the controls, or the untreated ones (Fig. 4).

Both of untreated and LA-treated EMF-exposed rats exhibited significantly upregulated hippocampal Sirt1 and Atg7 gene expression, compared to the control ones. In the meantime, LA-treated group had significantly downregulated hippocampal Sirt1 gene expression and significantly upregulated Atg7 gene expression, compared to the untreated group (Fig. 4).

Plasma ALT and AST activities were insignificantly changed among the different studied groups. Additionally, the initial and final body weights were insignificantly changed among the different studied groups (Table 4).

**Table 4:** Changes in plasma activities of ALT and AST (U/L), initial and final body weights (g) in the different studied groups.

	Control group	Multiple cell phones- exposed group	LA- treated-exposed group
Plasma ALT level (U/L)			
Mean ± SEM	26.98 ± 3.84	30.8 ± 4.19	23.57 ± 3.39
Plasma AST level (U/L)			
Mean ± SEM	59.1 ± 4.86	69.05 ± 6.06	71.86 ± 11.77
Initial body weight (g)			
Mean ± SEM	75.85 ± 1.62	78.23 ± 1.66	78. 54 ± 1.3
Final body weight (g)			
Mean ± SEM	200 ± 11.44	232 ± 14.15	233.15 ± 14.86

Non-significant changes were observed as regards liver enzymes and initial and final body weights.

### Correlation studies

Correlation studies in the three experimental rat groups revealed significant positive correlations between hippocampal glutamate and each of hippocampal Atg7 gene expression and percentage of change of latency in 5^th^ day. However, there were significant negative correlations between hippocampal MDA and each of hippocampal Atg7 gene expression and percentage of time spent in target quadrant in the test day (day 6). In addition, hippocampal Sirt1 gene expression had a significant negative correlation with percentage of time spent in target quadrant in day 6 in the different studied groups (Tables 5, 6 and 7).

**Table 5:** Correlations of hippocampal glutamate with hippocampal Atg7 gene expression, percentage of change of latency in day 5 in the different studied groups.

	Hippocampal Atg 7 gene expression	Percentage of change of latency in day 5
Hippocampal Glutamate		
Pearson correlation (r)	0.752	0.421
Significance (n)	< 0.001 (20)	< 0.05 (27)

**Table 6:** Correlations of hippocampal MDA Atg7 gene expressions, and % of time spent in target quadrant day 6 in the different studied groups.

	Hippocampal Atg 7 gene expression	% of time spent in target quadrant day 6
Hippocampal MDA		
Pearson correlation (r)	− 0.625	− 0.565
Significance (n)	< 0.02 (15)	< 0.002 (29)

**Table 7:** A correlation of hippocampal Sirt1 gene expression with % of time spent in target quadrant day 6, in the different studied groups.

	% of time spent in target quadrant day 6
Hippocampal Sirt1 gene expression	
Pearson correlation (r)	− 0.471
Significance (n)	< 0.05 (24)

### Histological results

**Figure 5:**
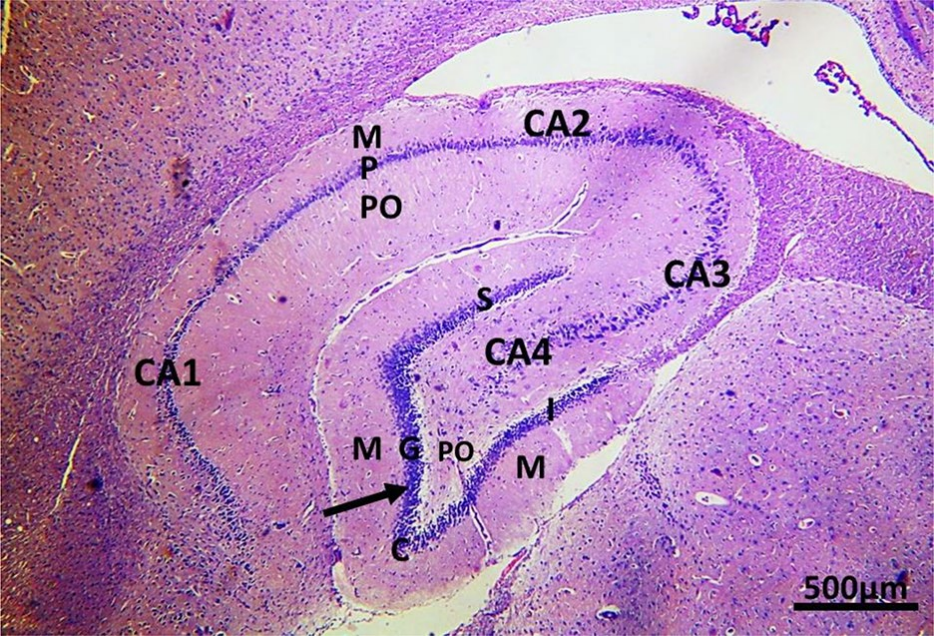
A photomicrograph of a sagittal section in the brain of control albino rat showing an overview of hippocampal formation: the dentate gyrus (↑) and the hippocampal proprius (proper). The hippocampus proper is formed of four regions of cornu ammonis (CA1, CA2, CA3, and CA4). Each is formed of three layers; molecular (M), pyramidal (P) and polymorphs layers (PO). The dentate gyrus appears as a dark V-shaped structure, with its open portion surrounding CA4 area of the hippocampus. The dentate gyrus is formed of three layers; Granule cell layer (G), molecular layer (M), and polymorphic layer (P). They are seen on both sides of CA4 layer, which extended toward the hilus of dentate gyrus. The molecular layer is seen continuous with that of the hippocampus in the depths of the hippocampal fissure. The supra-pyramidal blade (S) and infra-pyramidal (I) blade of the dentate gyrus are seen. The three layers are recognized in both blades. The crest (C) of dentate gyrus represents the meeting of both blades. Scale bar: 500 µm (H&E X 50).

Examination of H&E-stained sections of the brain of control group showed the hippocampal structure formed of the dentate gyrus and the hippocampal proprius (proper). The hippocampi proper consisted of four regions of cornuammonis (CA) (CA1, CA2, CA3, and CA4). The CA3 region showed a large pyramidal loosely packed neuron. Each region of hippocampus proper was formed of three layers; superficial molecular, middle pyramidal and deep polymorphic cell layers. Deeply stained interneurons were observed in both of molecular and polymorphic layers of the hippocampus. The dentate gyrus consisted of three layers; molecular layer, granular cell layer, formed of many granular cell neurons and polymorphic layer, which had densely stained interneurons. Both suprapyramidal and infrapyramidal blades had the same histological structure. Meeting of both blades occurs at the crest of dentate gyrus (Fig. 5).

**Figure 6:**
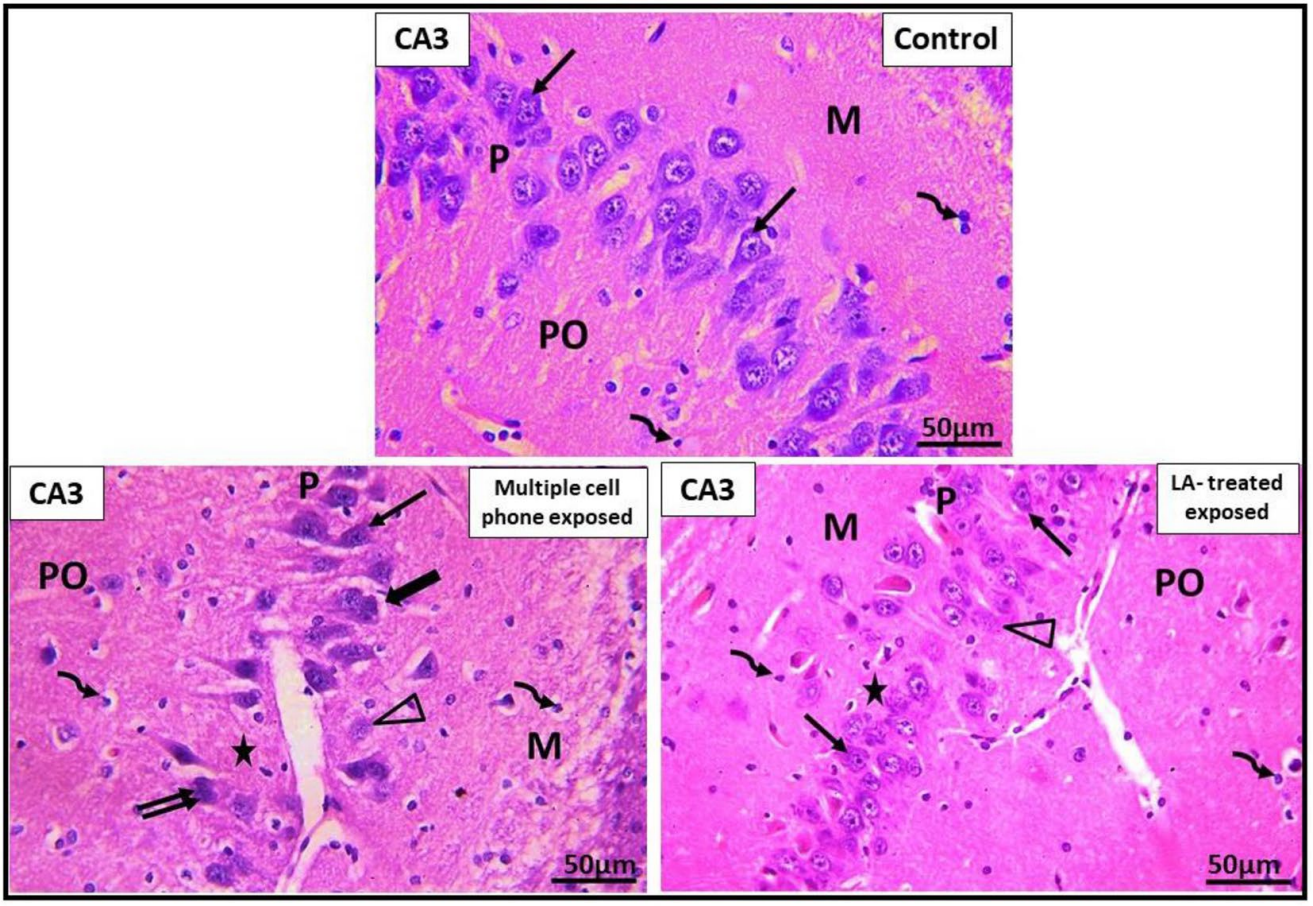
Photomicrographs of CA3 area of hippocampus of the different studied groups. CA3 area is formed of three layers; molecular layer (M), pyramidal (P) and polymorphic (PO) layers. Deeply stained interneurons (curved arrow) are seen in molecular and polymorphic layers. Pyramidal cells are seen with basophilic cytoplasm (↑) and vesicular nuclei. In the *control group*, pyramidal cells are arranged in compact layer with little neuropil between the cells. In *multiple cell phones- exposed group*, shrunken pyramidal cells with shrunken deeply stained basophilic cytoplasm (↑↑) are found surrounded with perineural space (thick arrow). Pyramidal cells with karyolitic pale stained nuclei (∆) and wide neuropil (*) are, also, observed between pyramidal cells. In *LA-treated exposed group,* some pyramidal cells are noted with pale stained nuclei (∆). Wide inter cellular spaces (*) are also noticed between pyramidal cells. Scale bars: 50 µ (H&E X400).

Examination of H&E-stained sections of CA3 area of hippocampus of the control group showed the pyramidal layer, which had crowded, evenly arranged pyramidal cells, with little neuropil in between. Most pyramidal cells were nearly the same size. Each cell had basophilic cytoplasm and contained a single rounded central largevesicular nucleus with prominent nucleolus. In multiple cell phones-exposed group, there were shrunken pyramidal cells with shrunken deeply stained pyknotic nuclei and deeply stained basophilic cytoplasm. Perineural spaces surrounding the degenerated pyramidal cells were observed. Pyramidal cells lost their even and regular arrangement. They were separated by wide neuropil, while some pyramidal cells were observed with karyolitic pale stained nuclei. In LA-treated group, occasional pyramidal cells were noted with pale stained nuclei and ill-defined margins. Wide intercellular spaces were noted between pyramidal cells (Fig. 6).

**Figure 7:**
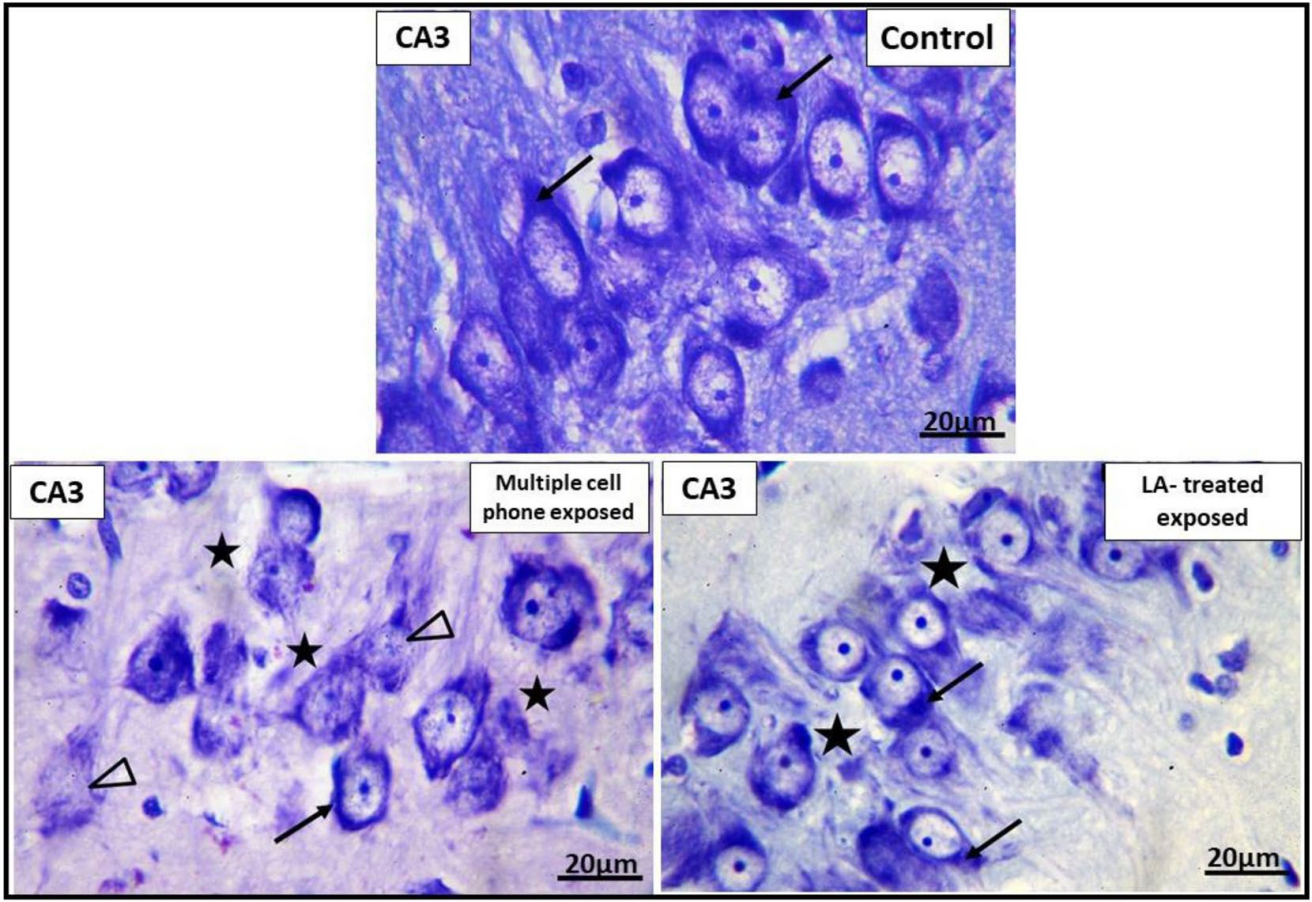
Photomicrographs of CA3 area of hippocampus of the different groups. Nissl granules are seen in cytoplasm of pyramidal cells (↑). In multiple *cell phones-Exposed group,* some pyramidal cells (Δ) are observed with ill- defined margins. Wide inter cellular spaces are noted between pyramidal cells (*). In *LA-treated exposed group*, wide inter cellular spaces (*) are found between pyramidal cells. Scale bar: 20 µm. (Toluidine blue X1000).

Examination of Toluidine Blue stained section of CA3 area of hippocampus of the control group showed obvious Nissl’s granules in the cytoplasm of pyramidal. In multiple cell phones-exposed rats, some pyramidal cells were observed with irregular outline. Wide intercellular spaces were present between pyramidal cells. Similarly, in LA-treated group, wide intercellular spaces were seen between pyramidal cells (Fig. 7).

**Figure 8:**
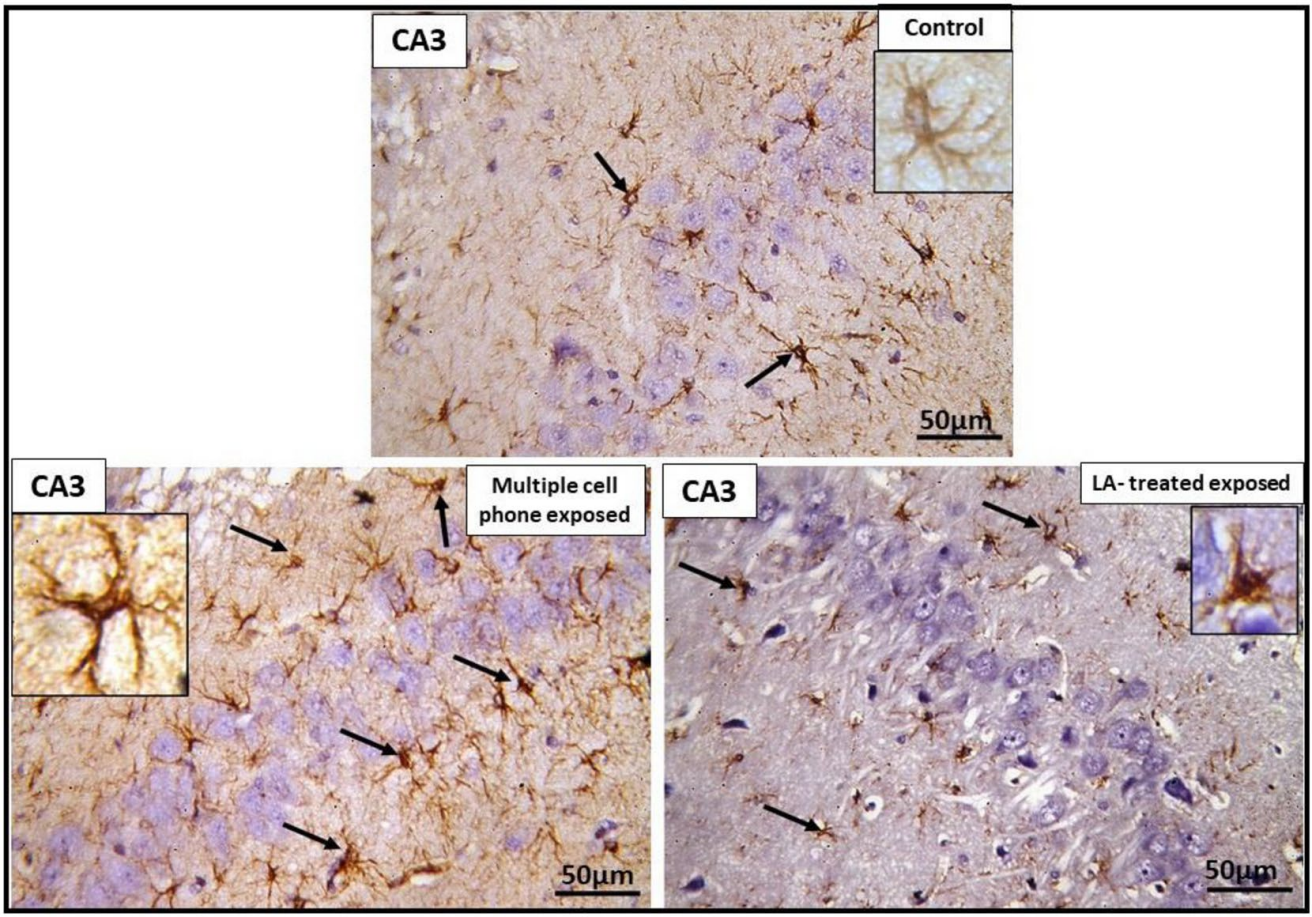
Photomicrographs of CA3 area of hippocampus of different groups, showing glial fibrillary acidic protein (GFAP) positive astrocytes (↑) in polymorphous and molecular layers. In *control group,* the inset shows astrocyte with its multiple processes. In *multiple cell phones-exposed group*, an apparent increase in number and intensity of GFAP positive astrocytes is noted. Glial fibers appear twisted, thickened and intensely stained compared to control group. Inset: astrocyte is seen with its multiple long processes that are intensely stained. In *LA treated exposed group*; GFAP positive astrocytes (↑) are seen in polymorphous and molecular layer. Insets: show astrocyte with their multiple processes. Scale bar: 50 µm (GFAP immune-staining X 400, inset X1000).

Examination of immune-stained sections of CA3 area of the control group revealed positive brownish reaction for GFAP in the cytoplasm of scattered glial cells in both polymorphic and molecular layers. In multiple cell phones- exposed group, an apparent rise of number and intensity of positively stained brownish astrocytes was observed in both polymorphic and molecular layers compared to that noted in the control rats. Astrocytes were present with their multiple long processes that frequently appeared twisted and thickened. In LA-treated group, positive brownish reaction for GFAP was observed in the cytoplasm of scattered astrocytes in both polymorphic and molecular layers similar to that in the control group (Fig. 8).

### Histomorphometric results

A significant decrease in Nissl`s granules density was observed in multiple cell phones-exposed group compared to the controls; meanwhile it was significantly elevated in LA-treated rats compared to either multiple cell phones-exposed group (Table 7).

The mean area percentage of GFAP positive astrocytes showed a significant rise of multiple cell phones-exposed group compared to the control ones. However, LA treatment resulted in a significant decline in the mean area percentage of GFAP compared to multiple cell phones-exposed rats (Table 8).

**Table 8:** Changes in Nissl granules density and area percentage of GFAP positive astrocytes in the different studied groups.

	Nissl’s granules density	Area % of GFAP positive astrocytes
Control group	97.1 ± 1.2	8.61 ± 0.5
Multiple cell phones-exposed group	68.3 ± 1.4^a^	19.32 ± 0.7^a^
LA-treated Exposed group	83.6 ± 1.3^b^	5.87 ± 0.4^b^

Statistical significance was determined using one-way ANOVA.

^a^Significance from the control group.

^b^Significance from multiple cell phone-exposed group.

## Discussion

This study demonstrated the possible beneficial effects of LA supplementation on hippocampal structure and function after long-term exposure to EMF, emitted from multiple cell phones to mimic real-life conditions. The current study used Morris water maze, as a memory test, which is frequently used to investigate visuospatial navigation, topographic disorientation, and motivational deficits. This maze examines the facilitations of content-dependent behaviour and reference memory in rats^31^.

In this study, rats exposed to multiple cell phones exhibit alterations in learning abilities, deduced from a significantly shorter time of latency to reach the platform by the fifth day of training. However, long-term multiple cell phones-exposure, herein, caused a reduced consolidation of spatial long-term memory, as evidenced by a significantly lowered time spent in the target quadrant than that of the control ones, tested by the probe test. In accordance, 45 min-exposure to a 2450 MHz–EMF caused deficits in spatial memory functions in Morris water maze^38^. The reduction of memory storage ability, which was induced by an extremely low-frequency magnetic field, was explained by its interaction with hippocampal neural activity^39^.

Similarly, Li et al.^40^ found that long-term microwave exposure impaired cognition function in rats and attributed that to the elevated neurotransmitters in the hippocampus, namely glutamate and hippocampal structural changes, such findings were also present in this study.

This cognitive impairment could be attributed to mitochondrial changes induced by EMF exposure, according to Gupta et al.^10^.

Several mechanisms tried to explain deleterious effects of EMF exposure on learning and memory; ROS production^5^, reduced melatonin level^41^, apoptosis^42^, modulation of the neurotransmitters release; also supported in the current study, higher plasma corticosteroids level^43^ and changes in sex hormones levels^44^.

The altered learning and reduced long-term memory in multiple cell phones-exposed rats could be considered novel findings as the other studies used EMF emitted from a single cell phone, and up to the authors’ knowledge, no present studies tested the effects of multiple cell phones as a source of EMF, particularly in puberty-adolescence transition time interval.

The results of Morris water maze, in the current study, were accompanied by higher hippocampal Ach levels in multiple cell phones-exposed rats. Interestingly, in the study of Abd El Rahman et al.^24^, there were high hippocampal Ach levels despite the absence of cognitive defects after single cell phone exposure. Thus, such cognitive changes could be dose-dependent effects as biological effects on neurons depend on exposure duration and the intensity of electromagnetic radiation^45,46^. In support, Hao et al.^47^ found that there was an initial reduction in task completion time then was normalized in the successive weeks of experimental period. Thus, they suggested that EMF may induce unusual temporary brain functioning in short term exposure conditions.

Correspondingly, Kumlin et al.^48^ implied that by the five-week EMF exposure, rats had a significant increase in performance in water maze learning and memory test. Studies also stated improvements in mice memory due to long-term exposure to high-frequency EMF. They suggested that EMF might represent a non-invasive, non-pharmacologic treatment against Alzheimer’s disease^49,50^.

Both learning and memory could be separated in certain cases, which was observed in Hashemi-Firouzi et al.^51^. These two processes depend on the nature of the interaction between Ach and its receptors in the hippocampus. Ach has differential effects on memory encoding and consolidation, favoring the encoding pathways. Ach promotes encoding new episodic memories in the hippocampus by enhancing the strength of afferent input relative to its feedback and activating intrinsic mechanisms for persistent spiking, resulting in the modification of synapses. During memory encoding, the cortex sends sensory inputs to the hippocampus, whereas the temporary memory is transferred back to the cortex for long-term storage during memory consolidation^52–54^.

Moreover, experimental and computer modeling studies demonstrated that Ach inhibits intrinsic pathways in the CA3 region of the hippocampus via the activation of muscarinic Ach receptors in interneurons, namely M2 and M4^52,55^. Meanwhile, Ach stimulates afferent projections, being a part of the encoding pathway and learning process, via nicotinic receptors α7^55,56^.

Therefore, Ach levels in the hippocampus are elevated during memory encoding, whereas they are low during memory consolidation^57^. This could suggest that higher Ach levels in multiple cell phones-exposed rats, in this study, stimulated the learning process or encoding, although it did not promote spatial memory consolidation. Thus, the diversity between hippocampal Ach levels and memory changes between the current study and previous studies may be explained by the presence of many factors controlling neural function after EMF exposure such as type, frequency, intensity of waves and exposure periods. Accordingly, increased EMF dosage induces severe consequences on brain neuronal function^45^.

Likewise, cognitive function changes induced by EMF exposure are explained, according to Li et al.^40^, by changes in hippocampal neurotransmitters. They suggested that accumulation of metabolic products could cause hippocampal damage after EMF long-term exposure (one month in their study). Such changes reflected an altered coordination between inhibitory and excitatory neurotransmitters, resulting in excitotoxic neuronal damage induced by excitatory neurotransmitter accumulation and/or neuronal excitability changes mediated by inhibitory neurotransmitters.

The increased hippocampal glutamate level in EMF-exposed rats, in the present study, partially explain the altered learning present in multiple cell phones-exposed rats, as N-Methyl-D-aspartate (NMDA) receptor enhances learning and memory^58^. To support this, hippocampal glutamate had a significant positive correlation with latency percentage change on the fifth day.

Furthermore, the histological changes in the hippocampus support the effects of higher EMF doses emitted from multiple cell phones. Degenerated pyramidal neurons of CA3, surrounded with large empty spaces, indicate cell retraction and lysis of the surrounding neuropil. These changes point to neurodegeneration and neuronal loss of adult hippocampal principal neurons in CA3, following long-term exposure to cell phone-EMF, herein. These findings are consistent with KV and NS^59^ and Elamin et al.^60^, who found that exposure to 900 MHz EMF causes severe alterations in the number and structure of hippocampal pyramidal neurons. This explains the memory deficits in multiple cell phones-exposed rats, in our study.

Correspondingly, we observed that long-term EMF exposure resulted in a marked increase in the number and activity of glial fibrillary acid protein (GFAP) immune-reactive astrocytes as well as the deep basophilic cytoplasm in pyramidal cells, similar to Afeefy et al.^61^. Astrocytes, glial cells in CNS, ensure neuronal survival through the maintenance of structural and metabolic support and regulation of the synaptic microenvironment. In addition, astrocytes maintain the blood–brain barrier, vascular reactivity, energy metabolism and reduced ROS levels^62^. They react to any CNS insult by overexpression of GFAP to preserve the neurons. This effect occurs through counteracting the inflammatory process, uptake of excito-toxic glutamate and production of neuroprotective adenosine and antioxidant glutathione^63^. This higher GFAP, present in multiple cell phones-exposed rats, may point out that glutamate level could not be at the neurotoxic level, herein.

As a result of EMF-exposure, the learning and memory impairment observed is associated with EMF-induced ROS production^5^, denoted by higher hippocampal MDA levels. Such changes match with Sharma and Shukla^64^. To support this, EMF was suggested to be detrimental or protective in neuronal response, depending on the dosage, frequency, and exposure period^45^.

Alternatively, LA treatment improved spatial memory after multiple cell phones exposure, similar to its action after single cell phone-exposure in our previously conducted study^24^. This memory improvement might be mediated by the decline in hippocampal Ach.

This spatial memory improvement in LA-treated rats is explained by the antioxidant effect of LA, implied by lowered hippocampal MDA level. To support this, a significant negative correlation was observed between hippocampal MDA levels and the percentage of time spent in the quadrant on day 6.

Mitochondria-derived ROS are released at physiological levels in normal signaling; meanwhile, pathological effects appear at higher levels. Lipoic acid, a mitochondria-directed antioxidant, was suggested to lower ROS production by enhancing Akt activation, and improving NO-mediated vasodilation^65^.

Additionally, LA-treated rats showed a less aggressive histopathological picture, compared to the untreated group, indicating the neuroprotective cytoprotective effects of lipoic acid. However, some histological derangements induced by EMF exposure in LA-treated rats were persistent. These changes were previously attributed to changes in brain structure, which will cause persistent damage if altered during the developmental period^66^.

EMF exposure causes deleterious effects on mitochondria, as in mitochondrial dysfunction associated with severe neurodegenerative conditions such as Alzheimer’s disease^67^.

Mitochondrial renewal via mitophagy has a role in determining cancer functionality and fate. Tumor cells rely on healthy mitochondria to promote their growth under changing microenvironmental stresses^68^. Recently, a growing consensus suggested that mitochondrial injury, mediated by chemotherapy and targeted agents, is attenuated due to increased mitophagy; this is associated with tumor therapeutic resistance and recurrence^69^. As autophagy maintains neuronal survival in many diseases^70^, the upregulated hippocampal Atg7 gene expression in multiple cell phones-exposed rats suggests a protective response to prevent severe neuronal damage. In accordance, autophagy was induced in the hippocampus with 835 MHz–EMF, emitted from cell phones for 12 weeks. Upregulated hippocampal Atg7 gene expression could be one of the various adaptive processes to EMF exposure^26^. This finding, herein, may point to the essential autophagy-mediated turnover of cellular constituents, eliminating dysfunctional or damaged mitochondria, to counteract the degeneration^71^.

Hence, autophagy could be a limiting mechanism against the injurious effects of oxidative stress, enhanced in multiple cell phones-exposed rats. To support this, there was a significant negative correlation between the Atg7 gene expression and the hippocampal MDA.

LA treatment resulted in upregulated hippocampal Atg7 gene expression to higher levels compared to the untreated exposed rats. It means upregulated Atg7 could be one of the cytoprotective mechanisms of LA to reduce the number of damaged mitochondria, which are ROS sources. To support this, there was a significant positive correlation between the hippocampal Atg7 and hippocampal glutamate, which points out the possible role of upregulated Atg7 in improving spatial memory in accordance with Guo et al.^72^.

SIRT1 is a crucial NAD-dependent deacetylase that has a role in maintaining mitochondrial function, promoting mitochondrial biogenesis, and regulating the autophagy-lysosome pathway^73^. SIRT1 directly interacts with the transcription factor FOXO3 and mediates its deacetylation. FOXO3 also regulates mitochondrial function and integrity by binding to the BNIP3 upstream promoter region, increasing BNIP3 expression and thus enhancing autophagy^74^.

Sirt1 is essential for normal cognitive function and synaptic plasticity in mice^75^, although its overexpression might induce memory deficits in transgenic mice^76^. The discovered upregulated hippocampal Sirt1 gene expression in multiple cell phones-exposed rats is attributed to the role of Sirt1 in the protection against chronic inflammation^77^. Similarly, Sirt1 expression was enhanced in the liver, resulting in a protective effect against high fat-induced hepatic steatosis^78^. The effect of Sirt1 is dependent on its level: at its low to moderate levels (about 3-to 8-fold), Sirt1 caused efficient protection against paraquat-induced cardiac stress and apoptosis in mice. As Sirt1 level in multiple cell phones-exposed rats, herein, was nearly 4-fold that of the controls, Sirt1 was protected against EMF-induced detrimental effects by keeping the integrity of hippocampal neurons by accelerating mitophagy of mitochondrial fission. In addition, Sirt1 enriches mitochondrial oxidative metabolism by reducing ROS generation through deacetylation of PGC-1α and nuclear factor kappa-light-chain-enhancer of activated B cells (NF-kB), this increases expression of antioxidant enzymes, namely glutathione peroxidase.

Furthermore, Sirt expression could enhance mitochondrial biogenesis through the Sirt1-PGC-1α axis in some mitochondrial abnormalities^79^. These Sirt1 actions may explain its higher gene expression in EMF-exposed rats herein.

LA treatment resulted in a significant elevation in the hippocampal Sirt1 gene expression, compared to the control group. In contrast, the level of Sirt1 gene expression declined in LA treated group, compared to the untreated group. The uppression of Sirt1 expression might be attributed to LA-ability to ameliorate oxidative stress-induced brain damage^80^. Correspondingly, Nicotinamide, a Sirt1 inhibitor, attenuated cognitive deficits in mice by promoting neuronal survival via the IGF-1pathway^81^, and through phosphorylation of tau^82^. To support this, the significant negative correlation between the percentage of time spent at the target quadrant and Sirt1 level may indicate a novel LA action, which is memory improvement through its cytoprotective and autophagy changes.

It is of value to highlight that males have long-lasting hippocampal long-term potentiation (LTP) contrasted with short-term potentiation in females. This sex-dependent LTP difference influences contextual learning^83^. Likewise, sex hormones contribute to the differential development and functioning of brain morphology, which are essential factors for learning and memory in the hippocampus and the amygdala^84^. Therefore, the sex difference in response to EMF has been demonstrated. The extent of damage to learning and memory in rats was previously found more in females than males^85^. Only male rats were used in this study to avoid any sex difference effects, particularly as we used rats that were exposed from early puberty to adolescence period.

From the results, it could be concluded that exposure to EMF emitted from multiple cell phones interfered with long-term memory. The enhanced Ach and glutamate levels are responsible for better learning ability, although acetylcholine alone may be responsible for the impairment of spatial memory. Histopathological changes in the hippocampus were evident and could be another cellular response that explains the upregulated hippocampal gene expression of Sirt1 and Atg7. In addition, lipoic acid treatment after EMF-exposure could improve learning ability as well as consolidation of memory, through elevating hippocampal glutamate and lowering hippocampal Ach in addition to ameliorating the oxidative stress proved by lowering hippocampal MDA level and upregulated hippocampal gene expression of Sirt1 and Atg7.

Further studies with longer duration of EMF-exposure, particularly in old age, are required to investigate the possible effects of overexpression of Atg7 and/or Sirt1 in hippocampal tissue as well as the further increase of glutamate level.

## Data Availability

The authors declare that all data underlying the findings described are fully available, without restriction, and from the time of publication, after contacting the corresponding author.
